# Risk of Lymph Node Metastasis and Feasibility of Endoscopic Treatment in Ulcerative Early Gastric Cancer

**DOI:** 10.1245/s10434-020-09153-7

**Published:** 2020-09-25

**Authors:** Ming-Han Ren, Xing-Si Qi, Yu-Ning Chu, Ya-Nan Yu, Yun-Qing Chen, Peng Zhang, Tao Mao, Zi-Bin Tian

**Affiliations:** 1 Department of Gastroenterology, The Affiliated Hospital of Qingdao University, Qingdao, Shandong China; 2 Department of Pathology, The Affiliated Hospital of Qingdao University, Qingdao, China

## Abstract

**Background:**

When the risk of lymph node metastasis (LNM) is considered minimal in patients with early gastric cancer (EGC), endoscopic submucosal dissection (ESD) is an effective alternative to radical resection. This study aims to estimate the feasibility of ESD for EGC with ulceration.

**Patients and Methods:**

We retrospectively reviewed data from 691 patients who underwent gastrectomy for EGC with ulceration. Subsequently, a stratification system for lesions was created based on the expanded ESD criteria, and the associations between the subgroups and the rate of LNM were analyzed.

**Results:**

LNM was confirmed in 16.5% (114/691) of patients. Univariate analysis demonstrated that age, sex, tumor size, macroscopic features, depth of invasion, tumor differentiation, Lauren type, lymphovascular invasion (LVI), and perineural invasion were associated with LNM. Multivariate analysis showed that LVI [odds ratio (OR) = 16.761*, P *< 0.001], SM1 invasion (OR = 2.159, *P *= 0.028), and SM2 invasion (OR = 3.230, *P *< 0.001) were independent risk factors for LNM. LNM occurred in undifferentiated mucosal tumors, with ulceration being 1.7% (2/116) when the lesion was smaller than 20 mm. Further stratification revealed that among lesions < 30 mm in size, undifferentiated tumors with SM1 invasion had a higher rate of LNM and a lower disease-free survival rate than differentiated tumors with SM1 invasion and tumors limited to the mucosal layer.

**Conclusions:**

Depth of invasion and LVI were strongly associated with LNM in ulcerative EGC. Endoscopic resection may be applicable for undifferentiated mucosal ulcerative EGC < 30 mm in size, and additional investigation is needed to evaluate its safety.

Early gastric cancer (EGC) is defined as carcinoma limited to the mucosa (T1a) or submucosa (T1b), regardless of presence of lymph node metastasis (LNM).^1^ Patients with EGC generally have extremely good prognosis after radical resection, and the 5-year survival rate is reported to be approximately 90%.^2^ When LNM develops, this survival rate decreases to less than 70%.^3^ The possibility of LNM makes gastrectomy the standard treatment for EGC. However, radical surgery is associated with various postoperative complications and a high mortality rate, as well as a decline in patient quality of life.^4^ Endoscopic submucosal dissection (ESD) is an effective alternative to surgical treatment for EGC when the risk of LNM is considered minimal.^5^ Based on the existing techniques, it is still difficult to accurately identify LNM perioperatively even if multidetector computed tomography (MDCT), magnetic resonance imaging (MRI), and endoscopic ultrasound (EUS) are applied.^6^,^7^

Since the risk of LNM needs to be minimal, ESD is recommended for use in well-or moderately well-differentiated EGC confined to the mucosa without ulceration and with lesion size equal to or smaller than 20 mm.^1^ The absolute indications are so strict that few patients are eligible for ESD. Subsequently, expanded indications were proposed for ESD that included ulcerated lesions, but the application was still limited to differentiated mucosal lesions with diameters smaller than 30 mm.^1^ In contrast with its use in non-ulcerative-type EGC, endoscopic submucosal resection is less commonly used in ulcerative-type EGC because of the higher risk of LNM in these tumors.^8^^–^10 Indeed, the factors affecting LNM have seldom been evaluated in patients with ulcerative EGC in previous studies.

This study, which involves a relatively large number of ulcerative-type EGC patients, aims to investigate the risk factors for LNM in patients with ulcerative lesions, identify the independent predictors of LNM in patients with ulcerative-type EGC, and verify whether the expanded ESD criteria are appropriate for the Chinese population.

## Patients and Methods

### Patients

We retrospectively reviewed all patients histologically diagnosed with EGC with ulceration who underwent gastrectomy with lymphadenectomy at the Affiliated Hospital of Qingdao University between June 2007 and December 2018. The selection criteria were (1) radical gastrectomy plus standard D1+/D2 lymph node dissection, (2) depth of tumor invasion confined to the mucosa or submucosa, and (3) ulceration confirmed by pathological examination. The exclusion criteria were (1) metastatic gastric cancer or multiple carcinomas, (2) lymphoma, (3) gastric stump carcinoma, (4) gross type neither 0–IIc (depressed) nor 0–III (excavated), (5) neoadjuvant chemotherapy or radiotherapy administered prior to surgery, and (6) other life-threatening diseases. A total of 691 ulcerative-type EGC patients were eligible for inclusion in this study.

### Data Collection

**Fig. 1:**
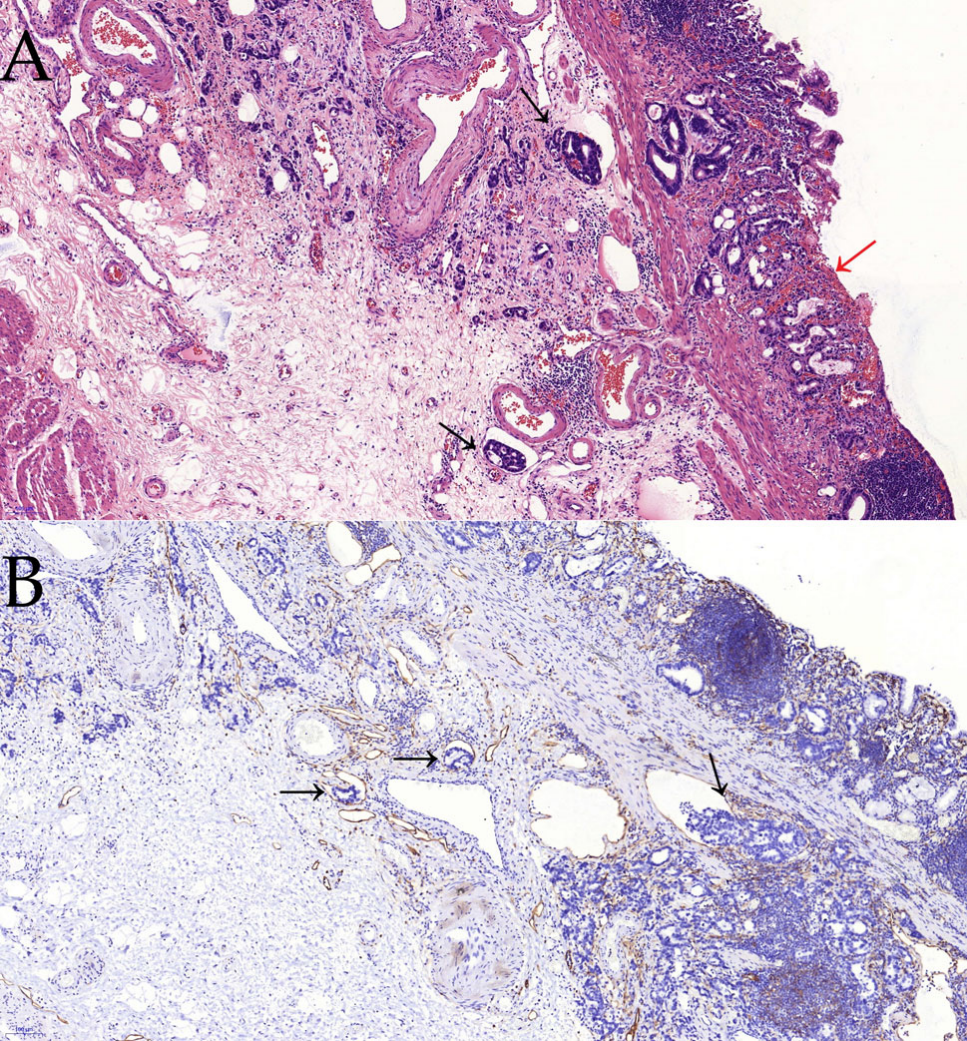
Pathological images (100 ×) of early gastric with ulcerative carcinoma: **a** lymphovascular invasion (black arrows) and ulceration (red arrow), stained by hematoxylin and eosin, and **b** lymphovascular invasion (black arrows) stained by D2-40

Based on location of cancer in the stomach and tumor histological characteristics, all surgeries (total or subtotal gastrectomy) involving lymph node dissection were performed in accordance with the 4th edition of the Japanese Gastric Cancer Association (JGCA) treatment guidelines.^1^ Tumor specimens were cut into 3-mm-thick slices, and histopathological examinations were completed individually by two expert pathologists. Presence of ulceration was defined as active ulceration or scarring from previous ulceration observed on histological examination, as shown in Fig. 1. To evaluate LNM, lymph nodes were sectioned into two pieces, and the sectioned surface was examined with hematoxylin and eosin staining. The pathological manifestation of lymphovascular invasion (LVI) is also shown in Fig. 1. Clinical data, endoscopic features, and pathological characteristics of the included patients were collected from the hospital information system of the Affiliated Hospital of Qingdao University.

We classified tumor locations into the upper, middle, and lower thirds of the stomach in accordance with the guidelines established by the JGCA. *Helicobacter pylori* infection status was determined by tests of biopsy specimens. Tumors were graded as small (≤ 20 mm), medium-sized (20–30 mm), or large (≥ 30 mm) to further analyze the ESD criteria. Based on the Paris endoscopic classification and the specimens we collected from ulcerative-type EGC, macroscopic features of EGC were divided into two subtypes: type 0–IIc (depressed) and type 0–III (excavated).^11^ Invasion depth was classified as M (intramucosal), SM1 (≤ 500 µm from the muscularis mucosae), or SM2 (> 500 µm from the muscularis mucosae). Moreover, we applied Nakamura’s classification to divide all tumors into differentiated and undifferentiated types.^12^ When multiple tumors were present, we classified the largest tumor with the same T stage or the tumor with the deepest invasion.^13^ In a previous study, researchers recommended that at least 15 lymph nodes should be examined after radical gastrectomy.^14^ In the present study, we divided the number of examined lymph nodes into two groups: < 15 and ≥ 15. Patients were divided into two groups according to surgical approach: total gastrectomy and subtotal gastrectomy.

The data consisted of age, sex, body mass index (BMI), incidence of hypertension, alcohol consumption, smoking history, carcinoembryonic antigen (CEA) level, *H. pylori* infection status, tumor location, lesion size, macroscopic type, depth of invasion, number of tumors, tumor differentiation, Lauren type, presence of lymphovascular invasion, presence of perineural invasion, number of lymph nodes examined, surgical approach, and LNM.

### Statistical Analysis

Statistical analyses were conducted with SPSS software (SPSS, version 23.0; Chicago, IL). Continuous variables were transformed into categorical variables, and categorical variables are presented as percentages of total number of cases. Since the mean age of the patients was 59.19 years, we selected 60 years as the cutoff value. According to the criteria for obesity, patients included in this study were divided into a nonobese group (BMI < 28 kg/m^2^) and an obese group (BMI ≥ 28 kg/m^2^). Differences between categorical variables were analyzed using a *χ*^2^ test or Fisher’s exact test. The risk factors considered significant on univariate analysis were subsequently included in multivariate logistic regression analyses to identify the independent variables associated with LNM. Then, lesions were stratified based on the expanded ESD criteria, and the associations between subgroups and the rate of LNM were analyzed. The 5-year overall survival (OS) and disease-free survival (DFS) rates of the subgroups were estimated. In all statistical analyses, *P* < 0.05 (two-sided) was considered statistically significant.

## Results

### Clinicopathological Characteristics

Among patients who underwent radical gastrectomy with lymph node dissection, 691 ulcerative-type EGC patients were eligible for inclusion in this study. Based on pathologic examinations, LNM was observed in 114 (16.5%) patients. At least 15 lymph nodes were examined in 533 (77.1%) patients, and fewer than 15 were examined in 158 (22.9%) patients. In total, 10.4% (72/691) of the patients underwent total gastrectomy, and 89.6% (619/691) of the patients underwent subtotal gastrectomy. There were 517 male patients and 174 female patients. The mean age was 59.19 years, ranging from 28 to 88 years; 348 patients were over 60 years of age, and an essentially equal number of patients (343 patients) were under 60 years of age.

The rate of *H. pylori* infection was 48.6% (336/691). Regarding location of tumor, 512 (74.1%) were in the lower third of the stomach, 161 (23.3%) were in the middle third, and 18 (2.6%) were in the upper third. In total, 405 (58.6%) lesions were small, while 174 (25.2%) lesions were medium-sized, and 112 (16.2%) lesions were large. The rates of LNM in these three groups were 12.1%, 20.1%, and 26.8%, respectively. Regarding macroscopic features, LNM occurred significantly more frequently in patients with type 0–III (excavated) than in patients with type 0–IIc (depressed) (*P* < 0.001).

Regarding depth of invasion, 291 (42.1%) had mucosal (M) tumors, 146 (21.1%) had SM1 tumors, and 254 (36.8%) had SM2 tumors. Undifferentiated lesions also occurred more frequently in patients with LNM than in those without LNM (*P* = 0.001). In terms of Lauren classification, we observed more LNM in diffuse-type (DT) and mixed-type (MT) tumors than in intestinal-type (IT) tumors (*P* < 0.05). Moreover, the LNM rates in patients with LVI (*P* < 0.001) and perineural invasion (*P* < 0.001) were significantly higher than those in patients without invasion. All the detailed clinicopathologic characteristics are presented in Table 1.

**Table 1:** Lymph node metastasis risk according to clinicopathologic characteristics

	Total (*n* = 691), *n* (%)	LNM negative (*n* = 577), *n* (%)	LNM positive (*n* = 114), *n* (%)	Univariate OR (95%CI)	*P* value
Age (years)					0.049
≤ 60	343 (49.6)	296 (51.3)	47 (41.2)	1	
> 60	348 (50.4)	281 (48.7)	67 (58.8)	1.502 (0.999, 2.256)	
Sex					0.015
Male	517 (74.8)	442 (76.6)	75 (65.8)	1	
Female	174 (25.2)	135 (23.4)	39 (34.2)	1.703 (1.105, 2.623)	
BMI (kg/m^2^)					0.142
≤ 28	630 (91.2)	522 (90.5)	108 (94.7)	1	
> 28	61 (8.8)	55 (9.5)	6 (5.3)	0.527 (0.221, 1.256)	
Smoking					0.176
Absence	354 (51.2)	289 (50.1)	65 (57.0)	1	
Presence	337 (44.8)	288 (49.1)	49 (43.0)	0.756 (0.504, 1.134)	
Drinking					0.179
Absence	441 (63.8)	361 (62.7)	79 (69.3)	1	
Presence	250 (36.2)	215 (37.3)	79 (69.3)	0.744 (0.483, 1.146)	
Hypertension					0.234
Absence	527 (76.3)	445 (77.1)	82 (71.9)	1	
Presence	164 (23.7)	132 (22.9)	32 (28.1)	1.316 (0.837, 2.068)	
*H. pylori* infection					0.187
Negative	355 (51.4)	290 (50.3)	65 (57.0)	1	
Positive	336 (48.6)	287 (49.7)	49 (43.0)	0.762 (0.508, 1.142)	
CEA					0.108
Negative	503 (72.8)	427 (74.0)	76 (66.7)	1	
Positive	188 (27.2)	150 (26.0)	38 (33.3)	1.423 (0.924, 2.191)	
Location					0.557
Upper	18 (2.6)	15 (2.6)	3 (2.6)	1	
Middle	161 (23.3)	130 (22.5)	31 (27.2)	1.192 (0.325, 4.375)	0.791
Lower	512 (74.1)	432 (74.9)	80 (70.2)	0.926 (0.262, 3.272)	0.905
Lesion size					< 0.001
Small (≤ 20 mm)	405 (58.6)	356 (61.7)	49 (43.0)	1	
Medium-sized (20–30 mm)	174 (25.2)	139 (24.1)	35 (30.7)	1.829 (1.137, 2.945)	0.012
Large (> 30 mm)	112 (16.2)	82 (14.2)	30 (26.3)	2.658 (1.590, 4.444)	< 0.001
Macroscopic type					< 0.001
0–IIc (depressed)	264 (38.2)	238 (41.2)	26 (22.8)	1	
0–III (excavated)	427 (61.8)	339 (58.8)	93 (77.2)	2.376 (1.488, 3.794)	
Number of tumors					0.913
Single	678 (98.1)	566 (98.1)	112 (38.2)	1	
Multitude	13 (1.9)	11 (1.9)	2 (1.8)	0.919 (0.201, 4.202)	
Invasion depth					< 0.001
M	291 (42.1)	271 (47.0)	20 (17.5)	1	
SM1	146 (21.1)	117 (20.3)	29 (25.5)	3.359 (1.826, 6.178)	< 0.001
SM2	254 (36.8)	189 (32.8)	65 (57.0)	4.660 (2.731, 7.953)	< 0.001
Differentiation					0.001
Differentiated	227 (32.9)	205 (35.5)	22 (19.3)	1	
Undifferentiated	464 (67.1)	372 (64.5)	92 (80.7)	2.304 (1.404, 3.782)	
LVI					< 0.001
Absence	593 (85.8)	543 (94.1)	50 (43.9)	1	
Presence	98 (14.2)	34 (5.9)	64 (56.1)	20.442 (12.314, 33.936)	
Perineural invasion					< 0.001
Absence	639 (92.5)	545 (94.5)	94 (82.5)	1	
Presence	52 (7.5)	32 (5.5)	20 (17.5)	3.624 (1.989, 6.603)	
Lauren’s type					0.035
Intestinal type	248 (35.9)	219 (38.0)	29 (25.4)	1	
Diffuse type	229 (33.1)	187 (32.4)	42 (36.8)	1.696 (1.017, 2.830)	0.042
Mixed type	214 (31.0)	171 (29.6)	43 (37.7)	1.899 (1.138, 3.168)	0.013
Surgical approaches					0.707
Subtotal gastrectomy	619 (89.6)	518 (89.8)	101 (88.6)	1	
Total gastrectomy	72 (10.4)	59 (10.2)	13 (11.4)	1.130 (0.598, 2.137)	
Lymph node examined					0.085
< 15	158 (22.9)	139 (24.1)	19 (16.7)	1	
≥ 15	533 (77.1)	438 (75.9)	95 (83.3)	1.587 (0.936, 2.691)	

*LNM* Lymph node metastasis, *OR* odds ratio, *CI* confidence interval, *BMI* body mass index, *H. pylori Helicobacter pylori*, *CEA* carcinoembryonic antigen, *M* tumor confined within the mucosal layer, *SM1* tumor invading the superficial (< 0.5 mm in depth) submucosa, *SM2* tumor invading the deep (> 0.5 mm in depth) submucosa, *LVI* lymphovascular invasion

### Univariate Analysis and Multivariate Analysis in EGC with Ulceration

As presented in Table 1, LNM was associated with age, sex, tumor size, macroscopic features, depth of invasion, tumor differentiation, Lauren type, presence of LVI, and presence of perineural invasion on univariate analysis. Multivariate analysis showed that depth of invasion and presence of LVI remained independent risk factors. Presence of LVI had the highest odds ratio (OR), followed by SM2 invasion and SM1 invasion. Details of independent risk factors are presented in Table 2.

**Table 2:** Multivariate logistic regression analysis of lymph node metastasis

Factor	OR (95% CI)	*P*-value
LVI	16.761 (9.986, 28.131)	< 0.001
Invasion depth		0.002
SM1	2.159 (1.086, 4.292)	0.028
SM2	3.230 (1.609, 5.293)	< 0.001

*LVI* Lymphovascular invasion, *SM1* invading the superficial (< 0.5 mm in depth) submucosa, *SM2* invading the deep (> 0.5 mm in depth) submucosa, *OR* odds ratio, *CI* confidence interval

### Stratification of Ulcerative EGC in Accordance with Expanded ESD Criteria

We stratified EGC with ulceration in accordance with the expanded ESD criteria and evaluated whether SM1 invasion, lesion size > 30 mm, and undifferentiated type were associated with higher probabilities of LNM. Lesions with SM2 invasion and LVI were excluded, and a total of 395 patients were included. As presented in Table 3, differentiated mucosal cancers with ulceration and size ≤ 30 mm, which were included in the expanded ESD criteria, had a relatively low rate of LNM. No LNM was observed in differentiated tumors ≤ 30 mm in size with SM1 invasion in our study. In the guidelines established by JGCA, endoscopic resection of these types of tumors is generally considered curative. Moreover, undifferentiated mucosal cancers with ulceration and size ≤ 30 mm also had a relatively lower incidence of LNM. However, undifferentiated tumors limited to the SM1 layer, regardless of size, had a relatively higher rate of LNM. In addition, we found a high proportion of LNM when tumor diameter exceeded 30 mm, regardless of differentiation and depth of invasion.

**Table 3:** Rate of lymph node metastasis according to stratification of pathological characteristics

Invasion depth	M		SM1	
Differentiation	D	UD	D	UD
Lesion size				
Small (≤ 20 mm)	3/77 (3.90)	2/116 (1.72)	0/39 (0.00)	**6/30 (20.00)**
Medium-sized (20–30 mm)	0/14 (0.00)	1/37 (2.70)	0/13 (0.00)	**2/20 (10.00)**
Large (> 30 mm)	**3/7 (14.29)**	**2/25 (8.00)**	**1/10 (10.00)**	**1/7 (14.29)**

Bold values indicate higher ratios of lymph node metastasis (over 8%), when compared with other values

*M* tumor confined within the mucosal layer, *SM1* tumor invading the superficial (< 0.5 mm in depth) submucosa, *D* differentiated type, *UD* undifferentiated type

### Statistical Analysis of Stratification Related to ESD Criteria

Based on the stratification described above, we found that the included lesions with diameter larger than 30 mm accounted for a small proportion of the total (49/395, 12.4%) but had a relatively high incidence of LNM (8–14.29%). Then, we decided to exclude lesions sized larger than 30 mm, and the remaining lesions were divided into four subgroups according to the depth of invasion and differentiation. Because differentiated mucosal ulcerative lesions ≤ 30 mm in size are included in the expanded ESD criteria, the *P*-values for the comparison between differentiated mucosal tumors and other subgroups are presented in Table 4, as are other details. In our study, only undifferentiated tumors with SM1 invasion were significantly different (*P* = 0.018) from differentiated mucosal tumors.

**Table 4:** Lymph node metastasis risk according to subgroups

	Total (*n* = 346), *n* (%)	LNM negative (*n* = 332), *n* (%)	LNM positive (*n* = 14), *n* (%)	*P*-value
Subgroup				
M + D	91 (26.3)	88 (26.5)	3 (21.4)	Reference
M + UD	153 (44.2)	150 (45.2)	3 (21.4)	0.823
SM1 + D	52 (15.0)	52 (15.7)	0 (0)	0.474
SM1 + UD	50 (14.5)	42 (12.7)	8 (57.1)	**0.018**

Statistically significant *P*-value is given in bold

*LNM* Lymph node metastasis, *M* tumor confined within the mucosal layer, *SM1* tumor invading the superficial (< 0.5 mm in depth) submucosa, *D* differentiated type, *UD* undifferentiated type

### Survival Analyses and Recurrence in Patients after Stratification

**Fig. 2:**
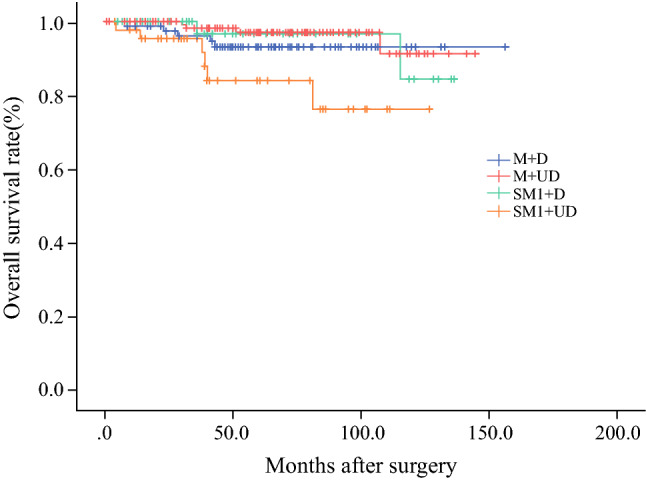
Overall survival in the subgroups. The 5-year overall survival rates in the M + D, M + UD, SM1 + D, and SM1 + UD were 89.6%, 96.0%, 94.7% and 76.1%, respectively

**Fig. 3:**
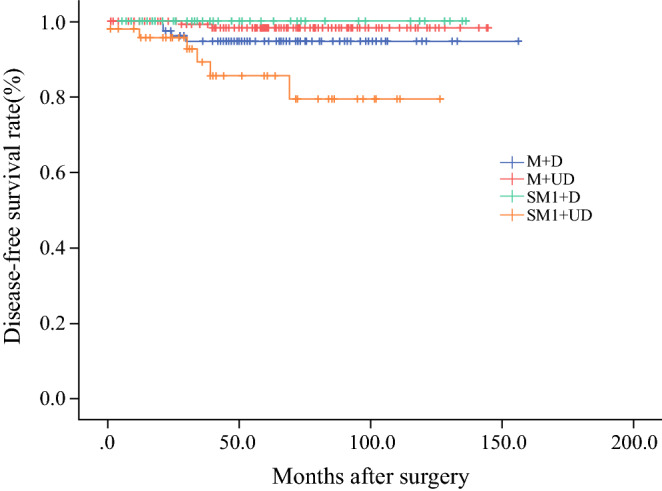
Disease-free survival in the subgroups. The 5-year disease-free survival rates of the M + D, M + UD, SM1 + D, and SM1 + UD group were 91.4%, 97.3%, 100%, and 76.1%, respectively

By the time of last follow-up visit for the 346 patients mentioned in Table 4, 21 (6.1%) had been lost to follow-up. Overall, the median follow-up period was 58 months. The cumulative 5-year OS rate of the 346 patients was 91.4%, and the 5-year DFS rate of these patients was 93.1%. Furthermore, the 5-year OS rates of the M + D group, M + UD group, SM1 + D group, and SM1 + UD group were 89.6%, 96.0%, 94.7%, and 76.1%, respectively (Fig. 2), and the 5-year DFS rates of the same groups were 91.4%, 97.3%, 100%, and 76.1%, respectively (Fig. 3).

Recurrence was observed in 3.69% (12/325) of patients. One patient experienced postoperative anastomotic recurrence, and 11 patients (3 patients with simultaneous metastasis were included) had distant metastasis, including metastasis of distant lymph nodes, bone, liver, lung, and peritoneum (Table 5). Overall, only five patients died of other illnesses or accidents. Two of these patients belonged to the M + UD group, and two belonged to the SM1 + D group. One belonged to the M + D group.

**Table 5:** Recurrence patterns according to subgroups

	Overall (*n* = 325)	M + D (*n* = 87)	M + UD (*n* = 143)	SM1 + D (*n* = 48)	SM1 + UD (*n* = 47)
Recurrence	12 (3.7%)	4 (4.6%)	2 (1.4%)	0 (0%)	6 (12.8%)
Anastomotic recurrence	1	0	1	0	0
Distant recurrence	11	4	1	0	6
Lung	2	1	0	0	1
Bone	4	2	0	0	2
Liver	4	1	1	0	2
Peritoneum	1	1	0	0	0
Distant lymph nodes	3	2	0	0	1

*M* tumor confined within the mucosal layer, *SM1* tumor invading the superficial (< 0.5 mm in depth) submucosa, *D* differentiated type, *UD* undifferentiated type

## Discussion

Currently, radical gastrectomy with D2 lymphadenectomy is considered a standard procedure for gastric cancer lesions localized with appropriate stages.^15^ In recent years, with the development of various auxiliary examinations and endoscopic techniques, an increasing number of EGCs can be diagnosed in the early stage. The long-term survival of EGC patients is generally supposed to be favorable, and the presence of LNM is the foremost prognostic factor.^16^,^17^ Since the rate of LNM was approximately 14% in previous studies, radical surgery may be excessive in the large proportion of patients without LNM.^18^^–^20 Gastrectomy often results in considerable postoperative complications, such as abdominal pain, nausea, vomiting, and dumping syndrome.^21^ As endoscopic resection is considered a curative form of resection, it is recommended as an alternative to radical surgery under specific conditions.^1^ Consequently, determining the presence or absence of LNM is crucial to the choice of treatment options.

EGCs with ulceration are common at the clinic. Since the finding of ulceration was considered to be a predictor of LNM occurrence,^9^ ulcerative tumors clinically diagnosed as differentiated T1a less than 30 mm in diameter without LVI were regarded as meeting the expanded indications established by the Japanese gastric cancer treatment guidelines. In recent years, there has been controversy over whether ulcers are associated with LNM.^22^^–^24 Few previous studies have assessed the risk of LNM in ulcerative EGC alone, but some researchers have investigated selected cases. A metaanalysis showed that, according to the expanded criteria for ESD, differentiated mucosal lesions < 30 mm with ulceration were associated with a minimal risk of LNM.^25^ A multicenter retrospective study in Japan, which included purely differentiated lesions and mixed but predominantly differentiated-type lesions, reported that LNM was not found in the 386 patients with ulcerative tumors who met the expanded indications.^26^ Kim et al.^27^ reported that no LNM was found either in mucosal and histologically differentiated ulcerative EGC regardless of tumor size or undifferentiated ulcerative carcinomas < 21 mm in diameter with limited submucosal invasion. In this study, we evaluated the clinicopathological features associated with LNM in patients with ulcerative EGC, and we stratified the lesions according to the endoscopic criteria to optimize the criteria for the Chinese population. To our knowledge, this study included the largest number of ulcerative EGC patients in a single-center retrospective study in China.

Overall, the incidence of LNM in ulcerative-type EGC patients was 16.5%, which is higher than the 12.5% reported by Lee et al.^10^ We found that deeper invasion and LVI are independent risk factors for LNM. In previous studies, depth of infiltration was also considered to be extremely relevant to LNM.^8^,^20^,^23^,^28^,^29^ Some studies reported that, although the mucosa showed an enrichment of blood capillaries, lymphatic ducts were only abundant in the deeper lamina propria and submucosa.^30^,^31^ This may explain the relationship between LNM and depth of invasion. LVI is the strongest risk factor for LNM occurrence, a finding that has been well documented in many studies.^32^ In addition, tumor size and differentiation also need to be considered. Our study also shows that tumor size and differentiation are associated with LNM, although they are not independent risk factors. Based on our results, these variables need to be taken into account when deciding whether ESD is appropriate. In our study, the classifications of differentiated and undifferentiated were based on Nakamura’s criteria. Notably, the mixed differentiated lesions were divided according to the predominant component (more than 50% of the composition), and this classification was recommended in the 2014 guidelines, which are still controversial.^1^ Kim reported that mixed differentiated lesions with undifferentiated components, which was an independent risk factor for LNM, had poor prognosis compared with purely differentiated types.^22^ A recent study reported that mixed differentiation was an independent risk factor for LNM in patients with EGC, and its OR was even higher than that of the purely undifferentiated type.^33^ The mechanisms involved in the mixed differentiated type need to be investigated in further studies. Since gross types are not mentioned in the ESD indications, they are often ignored in clinical practice. In our study, ulcerative lesions were divided into two categories, i.e., 0–IIc (depressed) and 0–III (excavated), and the rate of LNM in the latter was twice that in the former. We speculate that depth of lesions (or ulcerations) varies considerably, despite the pathological manifestations of ulcerations. In EGC with ulceration, depth of lesion invasion may have reached the lamina propria or submucosa, where there is an abundance of lymphatic vessels, when the macroscopic type is 0–III (excavated) type. Additionally, ulcers enrolled in this study included active and inactive ulcers. A previous study reported that presence of type 0–III (excavated) ulcers and incomplete ulcer healing were strongly associated with higher incidence of submucosal invasion.^34^ Similarly, Lee et al.^10^ showed that, for early gastric tumors, active ulcers are an LNM risk factor compared with healing and scarred ulcers but that elevated gross type was an independent risk factor for only differentiated-type gastric cancers. Since they can be approximately determined through endoscopy, gross appearance and activity of ulcers could be useful additional parameters to include in the preoperative indications for ESD, and a series of standardized descriptions are needed.

Subsequently, we stratified the ulcerative EGC patients without LVI according to the indicators commonly used in endoscopic therapy. As Table 3 shows, the patients who satisfied the expanded endoscopic criteria for ulcerative lesions had a LNM rate of 3.3%, which is different from the results observed under the same conditions in a Japanese study, where no LNM was found in 386 patients.^26^ A metaanalysis also showed that differentiated mucosal lesions < 30 mm with ulcerations had an LNM rate of 0.57%, which was relatively lower than the rate observed in our study.^25^ However, LNM was not observed (0/52) in patients with differentiated lesions smaller than 30 mm with superficial submucosal invasion and ulceration, which is somewhat different from the results of a metaanalysis that stated that the rate of LNM for lesions under the same conditions regardless of ulceration was 2.5%.^25^ Interestingly, we found that the incidence rate of LNM in patients with undifferentiated mucosal tumors with ulceration was 1.7% when the lesion was less than 20 mm and 2.7% when it was 20–30 mm. The rate was much lower than we had anticipated. Notably, when ulcerative-type EGC infiltrated the SM1 layer and was histologically undifferentiated, the rate of LNM increased significantly: 20% (6/30) in tumors less than 20 mm in size and 10% (2/20) in tumors more than 20 mm but less than 30 mm in size. Additionally, the rate of LNM was high (8–14.3%) when tumors > 30 mm in diameter were present. Our study suggests that, when the maximum diameter of the lesion is more than 30 mm, treatment other than endoscopic resection should be chosen. In a subsequent analysis, we divided lesions smaller than 30 mm into four groups according to depth of invasion (mucosal or SM1) and type of differentiation (differentiated or undifferentiated). The differentiated mucosal tumor group (M + D group), which was included in the indications in the expanded ESD criteria, was used as a reference to analyze whether rates of LNM were significantly different between the remaining three groups and the reference group (Table 4). Only the superficial submucosal differentiated tumor group (SM1 + UD group) had a rate significantly different from that in the reference group and was generally considered to not meet the indications for endoscopic resection. Interestingly, undifferentiated tumors limited to the mucosal layer, which meet the criteria for requiring surgery, had no significant difference in LNM compared with the differentiated mucosal group. In fact, the combination of mucosal invasion, histologically undifferentiated type, and ulceration has rarely been mentioned in previous studies. The guidelines state that endoscopically curative resection is only indicated for undifferentiated carcinomas with no ulceration and a diameter < 20 mm, which we believe are excessively strict criteria.^1^ Although undifferentiated lesions are considered to be highly invasive, tumors and ulcerations confined to the mucosal layer have little chance of reaching the lamina propria and submucosa, where the lymphatic ducts are thought to be abundant. In our study, mucosal undifferentiated EGCs < 20 mm in size with ulceration were found to be associated with a low incidence of LNM (1.7%, 2/116), and endoscopic treatments may be applicable to these types of tumors. Therefore, we suspect that undifferentiated mucosal EGC patients with presence of ulceration may have been overtreated in the past, especially patients with small lesions. However, based on our single-center study only, we cannot determine whether endoscopic treatments for undifferentiated mucosal lesions with ulceration are reasonable and adequate. As mentioned above, we need to consider more information when selecting treatments for ulcerated EGCs, such as gross types and ulcer activity, which should be noted in future investigations and clinical applications.

In the patients mentioned in Table 4, the 5-year OS and RFS rates were 91.4% and 92.5%, respectively. However, the 5-year OS and DFS rates in the SM1 + UD group were both 76.1%, which is a relatively poor outcome. With regard to long-term survival endpoints, radical surgery with D2 lymphadenectomy is the most effective therapy for EGC. However, postoperative complications cannot be ignored. Lee et al.^35^ reported that the incidences of postoperative complications in the laparoscopy-assisted distal gastrectomy group and open distal gastrectomy were 25.3% (220/1002) and 40.1% (232/629), respectively. It is important to seek new treatments as alternatives to surgery. Shichijo et al.^36^ reported that 94 patients with ulcerative lesions who met the ESD expanded criteria were confirmed to be alive 5 years after endoscopic resection. Yang’s study of undifferentiated EGC after noncurative endoscopic resection in Korea showed that the 5-year survival probability without lymph node metastasis or distant recurrence was 98.3% in patients without additional surgery.^37^ In Yang’s study, LVI, ulceration, submucosal invasion, and a positive vertical incision margin were independently associated with LNM and distant metastasis, and surgical resection was strongly recommended for patients with two or more risk factors. Compared with Li and Yang’s study, our study showed similarities in the occurrence of LNM and the 5-year DFS rate despite the different treatment methods (surgical treatment and endoscopic treatment).

In addition, we observed that male patients accounted for the majority of the patients. In previous studies, the development of gastric cancer was thought to be associated with lifestyle factors and androgen signal transduction pathways.^38^,^39^ In our study, lifestyle habits and the occurrence of hypertension were not significant risk factors for LNM. Another study reported that the effects of prolonged exposure to either ovaries or exogenous estrogen could reduce the risk of gastric cancer.^40^ However, female sex was associated with LNM on univariate analysis in our study. Moreover, a series of studies stated that female sex was significantly associated with the presence of LNM in EGC patients.^25^,^29^,^41^,^42^ The relationship between sex and LNM in EGC needs further investigation.

Several limitations to the present study should be acknowledged. First, this was a retrospective study at a single institution. Second, the reviewed data were from only surgically cured cases. Third, since complete histological characteristics were not available before specimens were obtained during gastrectomy or ESD, they may be difficult to include in the preoperative ESD criteria. A recent study reported that presence of histologic ulcers was unclear in some surgically resected EGC samples despite the definite endoscopic determination of an ulcer.^34^ Therefore, the comparison of postoperative pathological information with preoperative endoscopic reports is necessary for both ulcerative lesions and non-ulcerative lesions.

## Conclusions

Depth of invasion and LVI were strongly associated with LNM in ulcerative EGCs. When deciding whether ulcerative EGC is suitable for endoscopic resection, more parameters may need to be taken into consideration. Endoscopic resection may be applicable to undifferentiated mucosal ulcerative EGCs < 30 mm, although additional investigations of its safety are needed.
